# Very low-level prenatal mercury exposure and behaviors in children: the HOME Study

**DOI:** 10.1186/s12940-018-0443-5

**Published:** 2019-01-09

**Authors:** Nimesh B. Patel, Yingying Xu, Lawrence C. McCandless, Aimin Chen, Kimberly Yolton, Joseph Braun, Robert L. Jones, Kim N. Dietrich, Bruce P. Lanphear

**Affiliations:** 1 Faculty of Health Sciences, University of Lethbridge, 4401 University Drive, Lethbridge, Alberta T1K 3M4 Canada; 2 Cincinnati Children’s Hospital Medical Center, Department of Pediatrics, Division of General and Community Pediatrics, Cincinnati, OH USA; 3 Faculty of Health Sciences, Simon Fraser University, Burnaby, BC Canada; 4 Department of Environmental Health, University of Cincinnati, Cincinnati, OH USA; 5 Cincinnati Children’s Hospital Medical Center, University of Cincinnati College of Medicine, Department of Pediatrics, Division of General and Community Pediatrics, Cincinnati, OH USA; 6 Department of Epidemiology, Brown University, Providence, RI USA; 7 Centers for Disease Control and Prevention, Atlanta, GA USA; 8 Child and Family Research Institute, BC Children’s and Women’s Hospital, Vancouver, BC Canada

**Keywords:** Mercury, Methylmercury, Prenatal exposure, Behaviors, ADHD, Anxiety, Behavioral assessment system for children, BASC, Spence Children’s anxiety scale, SCAS

## Abstract

**Background:**

Mercury is toxic to the developing brain, but the lowest concentration associated with the development of behavior problems is unclear. The purpose of this study was to examine the association between very low-level mercury exposure during fetal development and behavior problems in children.

**Methods:**

We used data from 389 mothers and children in a prospective pregnancy and birth cohort study. We defined mean prenatal mercury concentration as the mean of total whole blood mercury concentrations in maternal samples collected at 16- and 26-weeks of gestation, delivery, and neonatal cord blood samples. We assessed parent-reported child behavior up to five times from two to 8 years of age using the Behavioral Assessment System for Children (BASC-2). At 8 years of age, we assessed self-reported child anxiety using the Spence Children’s Anxiety Scale (SCAS). We used multiple linear mixed models and linear regression models to estimate the association between mean prenatal mercury concentrations and child behavior and anxiety, respectively.

**Results:**

The median prenatal total blood mercury concentrations was 0.67 μg/L. Overall, we did not find statistically significant associations between mean prenatal mercury concentrations and behavior problems scores, but a 2-fold increase in mercury concentrations at 16-weeks gestation was associated with 0.83 point (95% CI: 0.05, 1.62) higher BASC-2 anxiety scores. Maternal and cord blood mercury concentrations at delivery were associated with parent-reported anxiety at 8 years.

**Conclusion:**

We found limited evidence of an association between very-low level prenatal mercury exposure and behaviors in children, with an exception of anxiety.

## Background

The neurotoxicity of mercury at high doses was established in congenital mercury poisonings in Japan and Iraq [1–4]. Methylmercury, the most common form of organic mercury, is of a particular concern because it can migrate through cell membranes, including the blood brain barrier, and bioaccumulate in tissues [5–7]. Low-level mercury exposure has been associated with deficits in intellectual abilities and externalizing behavior problems, but few studies have examined the impact on internalizing behaviors [5, 6, 8–19]. Reported effects of methylmercury exposure have only been consistent in older children and, in some cases, after adjustment for fish intake [5, 8, 10, 13, 18, 20]. Some investigators have found evidence for an apparent threshold of mercury exposure at 1 μg/g in maternal hair mercury concentrations, which is approximately equivalent to 4–5 μg/L in maternal blood [18, 21, 22]. But there is uncertainty about the effects of mercury at levels below the reported threshold which we defined as very-low.

We hypothesized that very low-level prenatal whole blood mercury concentrations (< 4–5 μg/L) would be associated with behavior problems among children from 2 to 8 years of age. In general, fetuses may be more susceptible to the adverse effects of exposure to environmental toxicants because they have rapidly developing organ system, different pharmacokinetics and higher exposure dose per body surface area compared to adults. Any disruption during pregnancy can significantly result in adverse outcomes in growth and development. The magnitude of adverse outcomes also depends on the timing of the exposure [early vs late pregnancy exposure] and these periods of heightened sensitivity are toxicant-specific. This makes it necessary for researchers to study and understand these periods of heightened vulnerability/window of vulnerability [23]. For mercury, these windows of vulnerability are not well studied/known. Hence, we also sought to identify windows of heightened vulnerability during fetal development using serial blood samples collected during pregnancy and at delivery.

## Methods

### Study design

We used data from a prospective pregnancy and birth cohort study, the Health Outcomes and Measures of the Environment (HOME) study, in Cincinnati, Ohio. The original study was designed to test and quantify the association between low-level prenatal and postnatal exposure to various environmental toxicants with the development of cognitive and behavioral problems in children. We enrolled pregnant women in the study from March 2003 to January 2006. Eligibility criteria at enrollment were: a) age ≥ 18 years, b) between 13 to 19 weeks of gestation, c) residing in a house built before 1978, d) not on a medication for seizure or thyroid disorders, and e) negative HIV status. Of 1263 eligible pregnant women, 468 (37%) agreed to participate in the study and 389 women remained in the study until delivery of a liveborn singleton infant. The details about the study cohort are described elsewhere [24].

### Measurement of mercury exposure

We collected whole blood samples from mothers at approximately 16 - and 26 weeks of gestation, and at the time of delivery. We also collected whole blood samples from children at birth (cord blood) and at the 2, 3, 4, 5, and 8 year visits. The whole blood samples were collected in EDTA vacutainer tubes that were lot tested for metal contamination, and stored at − 80 °C until shipment to the United States Centers for Disease Control and Prevention (CDC) for analysis. Total whole blood mercury was quantified using inductively coupled plasma mass spectrometry (ICP-MS) [25]. This multi-element analytical technique is based on quadruple ICP-MS technology. All analytical results were referenced to the National Institute of Standards and Technology (NIST) Standard Reference Materials (SRMs) along with two levels of quality control materials in each analytical run. The limit of detection (LOD) for total mercury was 0.2 μg/L. For results below the LOD (16, 19, 14, 16, and 8% for 16-week, 26-week, maternal blood samples at delivery, cord blood), we imputed a value of the LOD divided by square root of 2 [26].

### Assessment of children’s behaviors

Children’s behavior was assessed using the second edition of the Behavioral Assessment System for Children (BASC-2) Parent Rating Scale during follow-up visits when children were 2, 3, 4, 5, and 8 years. The BASC-2 is a reliable and valid assessment of a child’s adaptive and problem behaviors in community and home settings [27, 28]. The scale has four composite scores comprised of 12 clinical subscales. We examined the externalizing problems composite (hyperactivity and aggression subscales), internalizing problems composite (anxiety, depression, and somatization), the behavior symptoms index (BSI) composite (aggression, hyperactivity, depression, atypicality, withdrawal, and attention problems); and adaptive skills composite (activity of daily living, adaptability, social skills, and functional communication). We used T-scores based on combined-sex norms for analysis that are normalized to a mean of 50 and standard deviation (SD) of 10. A score > 60 is considered “at-risk behavior,” with the exception for adaptive skills and corresponding subscales, in which a score < 40 is non-optimal. We were primarily focused on examining the relationship of prenatal mercury with composite behaviors (i.e., externalizing problems, internalizing problems, BSI, and adaptive skills), but we also examined the associations with ADHD (i.e., aggression, hyperactivity, attention) and anxiety subscales as prior studies found that prenatal mercury was associated with these specific behaviors [8, 12, 16, 18].

We also assessed anxiety in children using the self-reported Spence Children’s Anxiety Scale (SCAS) when the children were about 8 years of age. The SCAS assesses six domains including generalized anxiety, panic/agoraphobia, social phobia, separation anxiety, obsessive-compulsive disorder, and physical injury fears [29]. A trained examiner oriented the child to the use of a Likert scale before reading each question to the child and allowing them to circle their answer on the score sheet. We used age- and sex-standardized scores for the analysis.

All assessments for the study were conducted in a standardized clinic setting to minimize distractions. The examiners were blinded to maternal and child mercury concentrations.

### Covariates

Our selection of potential confounders was guided by other research studies [16, 18, 30]. We included variables as potential confounders that were associated with both mercury exposure and behavior changes in children but were not known to be on the causal pathway. Fish intake, a source of methylmercury exposure as well as nutrients that are beneficial for brain development, can be an important confounder of the effect of mercury on child behavior [5, 31]. At the baseline visit (about 20 week of gestation), we asked the mothers about fish or shellfish consumption during pregnancy from the estimated date of conception until the survey was taken. We categorized the frequency of reported fish intake as: none, < once per month, 1 to 3 times per month, and > once per week.

We examined other variables as potential confounders, including maternal age at delivery (≤30 years and > 30 years), maternal ethnicity (white and nonwhite), annual household income (≤ $40,000 and > $40,000), maternal education (completed and not completed bachelor’s degree), marital status (married/living with partner and unmarried/living alone), and child sex. Maternal age at delivery and annual household income were categorized based on the frequency distribution of the variables. We also adjusted for the caregiving environment using the Home Observation for Measurement of the Environment (HOME) Inventory conducted during a 12-month home visit [32]. We categorized HOME Inventory scores as < 40 and ≥ 40 based on the distribution of the scores. We also measured maternal depression using the second edition of the Beck Depression Inventory (BDI-II) at 20 weeks of gestation [33]. We further classified the scores as minimally depressed (BDI-II score ≤ 13) and greater than minimally depressed (BDI-II score > 13). In addition, we examined whether children’s blood lead and mercury concentrations, and maternal pregnancy concentrations of serum cotinine, polychlorinated biphenyls (PCBs), urinary bisphenol A (BPA) were confounders or effect modifiers of the associations between prenatal mercury concentrations and child behavior.

### Statistical analysis

We defined mean prenatal mercury concentrations as the mean of mercury measured in whole blood collected at 16 and 26 weeks gestation, at delivery from the mothers, and in cord blood from the infants. We used average of the log_2_-transformed mercury concentrations to reduce the influence of outliers. We compared 389 women and their children’s (original sample) demographic characteristics with 320 dyads who had at least one blood mercury and BASC-2 data. We compared the 320 dyads with at least one mercury and behavior assessment with 69 dyads not included in the analyses because of missing either mercury and behavior assessment. We also stratified mean prenatal mercury concentrations and child BASC-2 scores of 226 dyads who were followed at the 8-year visit by demographics and by maternal and child characteristics.

We examined the associations of prenatal mercury with BASC-2 composite externalizing problems, internalizing problems, and BSI, and adaptive skills using linear mixed models with an unstructured covariance matrix. The linear mixed models account for the correlated nature of the repeated observations. Thus, the coefficient from a linear mixed model would represent an average increase in mean BASC-2 scores for every 2-fold increase in mercury concentrations.

We constructed linear mixed models for composite scores. First, we constructed unadjusted models. Then we added fish intake in the unadjusted models to test for confounding with whole blood mercury. Next, we added participants’ demographics and characteristics, child blood lead concentrations, maternal serum cotinine concentrations with and without child mercury concentrations to the unadjusted models and called this multivariable model 1. We added fish intake to the multivariable model 1 and call this model multivariable model 2. As mentioned before, the choice of the inclusion of the variables in the models was based on the criteria of confounders (associated with both mercury exposure and behavior changes in children but were not known to be on the causal pathway) and also guided by previous research [16, 18, 30]. The findings of the different regression models are presented in Fig. 2. Multivariable model 2 had the lowest Akaike’s information criterion (AIC) values compared to multivariable model 1 and considered this our primary regression model. We then constructed primary regression model for all corresponding BASC-2 subscales. We used locally weighted scatterplot smoothing (LOESS) analysis to examine the shape of the relationship for blood mercury and behaviors in adjusted models. The relationships appeared to be linear (Fig. 3). We used statistical software SAS 9.3 and R for all analyses and GraphPad Prism to prepare some graphs for the manuscript.

### Secondary analysis

We conducted secondary analyses to test for evidence of a developmental window of vulnerability for behaviors that were associated with mean prenatal mercury concentrations. We constructed a series of regression models for 16- and 26 weeks gestation, at delivery, cord and mean prenatal mercury concentrations with and without including cord blood concentrations. Not only anxiety, we also examined the association of prenatal mercury concentrations and other composite scores/subscales at 8-years of age. We examined anxiety at 8-years of age in more depth because there is some evidence suggesting an association between prenatal mercury exposure and anxiety [16, 34]. Although this was at higher prenatal mercury levels, it was important for us to examine if such association exists at very low – yet representative – levels of prenatal mercury exposure as we found some evidence of positive association between mean prenatal mercury concentrations and BASC-2 anxiety subscale. So, we also examined the Pearson correlation coefficient between 8-year BASC-2 anxiety subscale and 8-yr SCAS anxiety total scores. Finally, we examined the associations of prenatal mercury concentrations with parent reported BASC-2 anxiety subscale and self-reported SCAS total scores at 8-years using multivariable linear regression models.

Additionally, we examined models stratified by child sex and maternal race/ethnicity. We checked for two-way interactions by adding interaction terms for child age, child sex, maternal race/ethnicity, child blood lead concentrations, maternal cotinine concentrations, and PCBs with mercury at each prenatal visit. Finally, we examined the association between mean of post-natal child blood mercury concentrations and behavior scores in children.

## Results

Out of 468 enrolled pregnant women in the study, 67 (14.3%) dropped out of the study before delivery, 12 (2.6%) delivered twins or stillborn children, and 389 (83.1%) delivered live singleton infants. The 320 (82.2%) dyads who had at least one mercury and one behavior assessment were similar to the original sample of 389 mothers and their singleton children (Table 1). However, the 320 dyads were older (>30 years), more educated (completed Bachelor’s degree), less depressed (BDI≤13), had a higher proportion being white, had higher income at baseline (> $40,000) and higher HOME score (≥40), compared to the 69 dyads not included in the analyses because of missing either mercury or behavior assessment (Table 1).

**Table 1:** Characteristics of all mother-child dyads,those with at least one prenatal mercury and one BASC-2 behavior data, and dyads not included in the analyses due to missing either mercury or behavior assessment data [no. (%)]

Variable	Women and singleton children (*n* = 389)	Women and singleton children with at least one mercury and behavior measurement (*n* = 320)	Dyads not included due to missing either mercury or behavior assessment data (*n* = 69)
Maternal age at delivery			
≤ 30 years	207 (53.2)	162 (50.6)	45 (65.2)
> 30 years	182 (46.8)	158 (49.4)	24 (34.8)
Missing	0	0	0
Maternal ethnicity			
White	237 (61.7)	206 (64.4)	31 (48.4)
Others	147 (38.3)	114 (35.6)	33 (51.6)
Missing	5	0	5
Midpoint value of income category at baseline			
> $ 40,000	231 (60.2)	200 (62.5)	31 (48.4)
≤ $ 40,000	153 (39.8)	120 (37.5)	33 (51.6)
Missing	5	0	5
Maternal education			
Completed Bachelor’s degree	191 (49.7)	170 (53.1)	21 (32.8)
Not completed Bachelor’s degree	193 (50.3)	150 (46.9)	43 (67.2)
Missing	5	0	5
Maternal marital status at baseline			
Married or living with partner	300 (78.1)	249 (77.8)	51 (79.7)
Unmarried or living alone	84 (21.9)	71 (22.2)	13 (20.3)
Missing	5	0	5
Child sex			
Male	181 (46.5)	145 (45.3)	36 (52.2)
Female	208 (53.5)	175 (54.7)	33 (47.8)
Missing	0	0	0
HOME score (at 12 months)			
≥ 40	219 (65.6)	204 (67.6)	15 (46.9)
< 40	115 (34.4)	98 (32.4)	17 (53.1)
Missing	55	18	37
Maternal depression (BDI-II) at 20 weeks			
Minimal depression (≤13)	296 (77.7)	254 (80.1)	42 (65.6)
Mild, moderate or severe depression (> 13)	85 (22.3)	63 (19.9)	22 (34.4)
Missing	8	3	5
Fish intake			
Not at all	57 (14.9)	48 (15.0)	9 (14.1)
Less than once a month	114 (29.8)	94 (29.5)	20 (31.2)
1 to 3 times a month	132 (34.4)	108 (33.9)	24 (37.5)
More than once a week	80 (20.9)	69 (21.6)	11 (17.2)
Missing	6	1	5

**Fig. 1:**
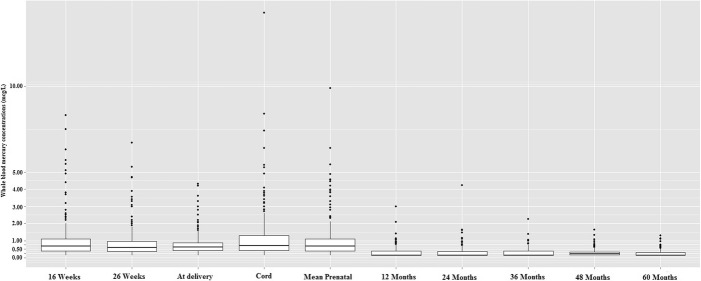
Box plots of maternal and child whole blood total mercury concentrations (μg/L) in the HOME study. “16 weeks”, “26 weeks”, and “At delivery” represents maternal blood mercury concentrations at 16-weeks, 26-weeks of gestation, and at delivery, respectively. “Cord” represents cord blood mercury concentrations at delivery. “Mean prenatal” represents mean of maternal mercury concentrations at 16 weeks, 26 weeks, delivery and cord blood mercury concentrations. “12 months”, “24 months”, “36 months”, “48 months”, and “60 months” represent child blood mercury concentrations at 1, 2, 3, 4, and 5 years of age

The arithmetic mean (±SD) and median prenatal mercury concentrations were 0.95 ± 1.02 and 0.67 μg/L, respectively. The median 16- weeks, 26-weeks, at delivery and cord blood mercury concentrations were 0.68, 0.60, 0.63, and 0.70 μg/L, respectively (Fig. 1). The mean T-scores and the SDs of the BASC-2 composite scores are normal (Table 2). Mean prenatal mercury concentrations were about 3.5 times higher in mothers who consumed fish more than once a week compared to those who did not consume fish (Table 2).

**Table 2:** Mean prenatal whole blood total mercury concentrations^a^ (μg/L) and child BASC-2 scores by characteristics

Characteristics	N (%) at 8 year visit^b^	Mean prenatal mercury levels (mean ± SD)	BASC-2 Externalizing score (mean ± SD)	BASC-2 Internalizing score (mean ± SD)	BASC-2 Behavior Symptoms Index (mean ± SD)	BASC-2 Anxiety (mean ± SD)
All participants	226 (100)	0.90 ± 1.00	49.3 ± 9.5	48.0 ± 8.9	49.6 ± 9.1	49.5 ± 9.7
Maternal age at delivery						
≤ 30 years	124 (54.9)	0.73 ± 0.58	49.8 ± 10.2	46.8 ± 8.7	49.6 ± 9.1	48.2 ± 9.7
> 30 years	102 (45.1)	1.10 ± 1.31	48.7 ± 8.5	49.6 ± 8.9	49.6 ± 9.1	51.1 ± 9.6
Maternal ethnicity						
White	135 (59.7)	0.95 ± 1.16	49.1 ± 8.5	48.7 ± 9.1	49.2 ± 8.6	50.6 ± 10.0
Others	91 (40.3)	0.81 ± 0.65	49.6 ± 10.8	47.1 ± 8.5	50.2 ± 9.7	47.9 ± 9.1
Midpoint value of income category at baseline						
> $ 40,000	131 (58.0)	1.04 ± 1.22	48.4 ± 8.1	48.7 ± 8.7	48.8 ± 8.8	50.5 ± 9.4
≤ $ 40,000	95 (42.0)	0.70 ± 0.47	50.6 ± 11.0	47.2 ± 9.1	50.7 ± 9.4	48.1 ± 10.0
Maternal education						
Completed Bachelor’s degree	105 (46.5)	1.13 ± 1.33	48.4 ± 7.7	48.3 ± 8.9	48.8 ± 8.7	50.5 ± 10.0
Not completed Bachelor’s degree	121 (53.5)	0.69 ± 0.47	50.1 ± 10.7	47.8 ± 9.0	50.3 ± 9.4	48.7 ± 9.5
Marital status at baseline						
Married or living with partner	164 (72.6)	0.96 ± 1.11	48.9 ± 8.5	48.4 ± 8.6	49.1 ± 8.7	50.0 ± 9.3
Unmarried or living alone	62 (27.4)	0.74 ± 0.53	50.4 ± 11.7	47.1 ± 9.6	50.8 ± 10.0	48.1 ± 10.8
Child sex						
Male	99 (43.8)	0.84 ± 0.74	51.0 ± 9.8	46.7 ± 8.3	50.7 ± 9.2	47.7 ± 8.7
Female	127 (56.2)	0.94 ± 1.15	48.0 ± 9.0	49.1 ± 9.2	48.8 ± 9.0	50.9 ± 10.3
HOME score (at 12 months)						
≥ 40	131 (62.4)	1.06 ± 1.23	48.5 ± 8.5	48.4 ± 8.5	48.7 ± 9.0	50.3 ± 9.4
< 40	79 (37.6)	0.68 ± 0.41	50.8 ± 10.4	48.3 ± 9.8	51.1 ± 9.1	49.1 ± 10.4
Maternal depression (BDI-II) at 20 weeks						
Minimal depression (≤ 13)	174 (77.7)	0.98 ± 1.10	48.1 ± 8.8	47.3 ± 8.5	48.5 ± 8.7	49.3 ± 9.6
Mild, moderate or severe depression (> 13)	50 (22.3)	0.61 ± 0.37	53.7 ± 10.7	50.8 ± 9.9	53.5 ± 9.4	50.3 ± 10.2
Fish intake						
Not at all	32 (14.2)	0.40 ± 0.28	47.6 ± 9.9	45.1 ± 8.2	47.6 ± 9.1	47.9 ± 10.1
Less than once a month	64 (28.3)	0.64 ± 0.53	47.9 ± 8.6	48.0 ± 9.2	48.8 ± 9.1	49.1 ± 9.3
1 to 3 times a month	80 (35.4)	1.00 ± 0.99	49.8 ± 8.8	48.6 ± 8.8	50.1 ± 8.9	49.9 ± 9.8
More than once a week	50 (22.1)	1.38 ± 1.41	51.4 ± 10.9	49.2 ± 8.9	51.1 ± 9.2	50.5 ± 9.9

^a^Mean prenatal mercury concentrations defined as a mean of maternal mercury concentrations at 16-weeks, 26-weeks of gestation, delivery, and cord blood mercury concentrations

^b^Participants at 8-year visit with at least one mercury measurement and all BASC-2 data

We found that cord blood mercury concentrations were strongly correlated with maternal mercury concentrations at 16 weeks (Pearson *r* = 0.71), 26 weeks (Pearson *r* = 0.77), and delivery (Pearson *r* = 0.82) with *p*-value of < 0.001 for all correlation coefficients (data not shown).

**Fig. 2:**
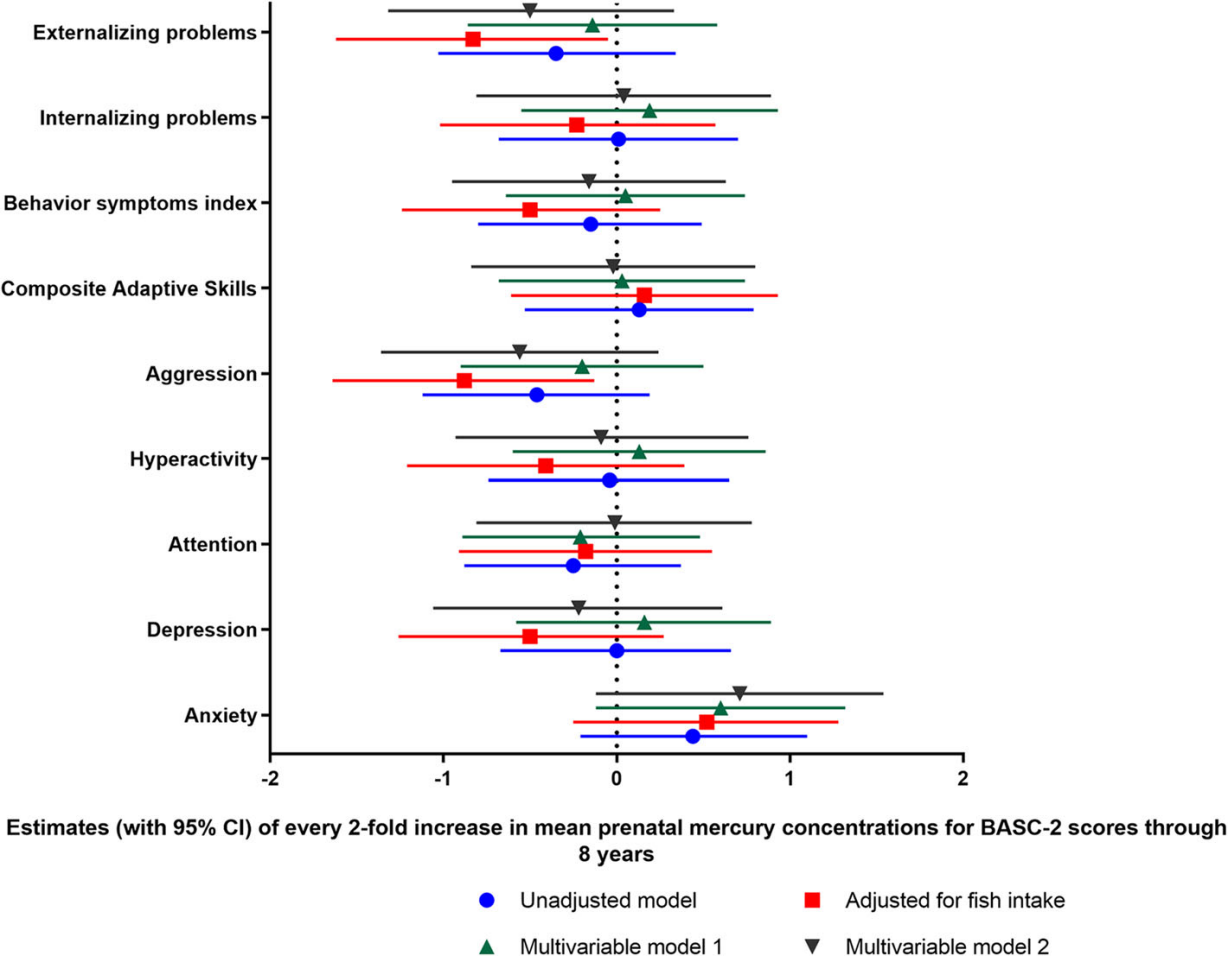
Comparison of different regression models on association between prenatal mercury [log_2_-transformed] concentrations and BASC-2 scores through 2–8 years. Mean prenatal mercury concentrations defined as a mean of maternal mercury concentrations at 16-weeks, 26-weeks of gestation, delivery, and cord blood mercury concentrations. Unadjusted model was not adjusted for any variables. “Adjusted for fish intake” model was adjusted only for fish intake. Multivariable model 1 was adjusted for maternal age at delivery (≤ 30 years and > 30 years), maternal ethnicity (white and others), annual household income (> $40,000 and ≤ $40,000), maternal education (Completed Bachelor’s degree and not completed bachelor’s degree), marital status at baseline (Married/living with partner vs. unmarried/living alone), HOME score (≥ 40 or < 40), maternal depression during pregnancy (≤ 13 and > 13), child sex, child blood lead concentrations (mean of child blood lead levels at 12, 24, 36, 48, and 60 months), pre-natal serum cotinine concentrations (mean of 16 weeks, 26 weeks, birth and cord levels) and post-natal child blood mercury concentrations (mean of child blood mercury levels at 2, 3, 4, 5 years). Multivariable model 2 was adjusted for all the variables included in model 1 and fish intake

**Fig. 3:**
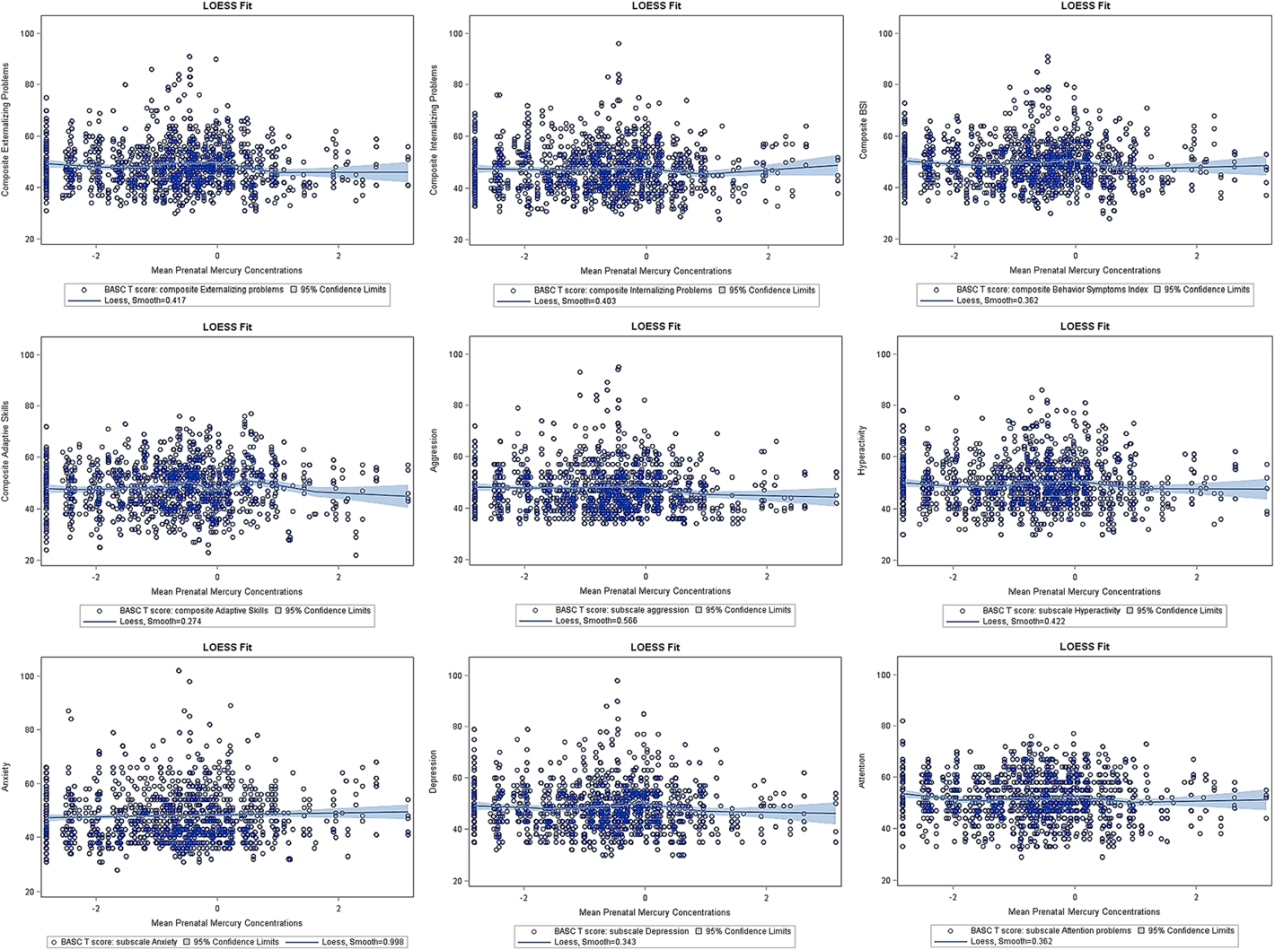
Locally weighted scatterplot smoothing (LOESS) plots for the relationship between mean prenatal [log_2_-transformed] mercury concentrations and BASC-2 scores through 2–8 years. Mean prenatal mercury concentrations defined as a mean of maternal mercury concentrations at 16-weeks, 26-weeks of gestation, delivery, and cord blood mercury concentrations. Solid blue lines represent mean regression lines, gray bands are 95% CIs, and dots are raw data. The relationship was smoothed using a value based on lowest AICC

Multivariable model 2 had the lowest AIC value and hence selected as the primary regression model (Fig. 2). Locally weighted scatterplot smoothing (LOESS) analyses revealed approximately linear relationships between log_2_-transformed prenatal mercury concentrations and BASC-2 behavioral outcomes (Fig. 3).

**Fig. 4:**
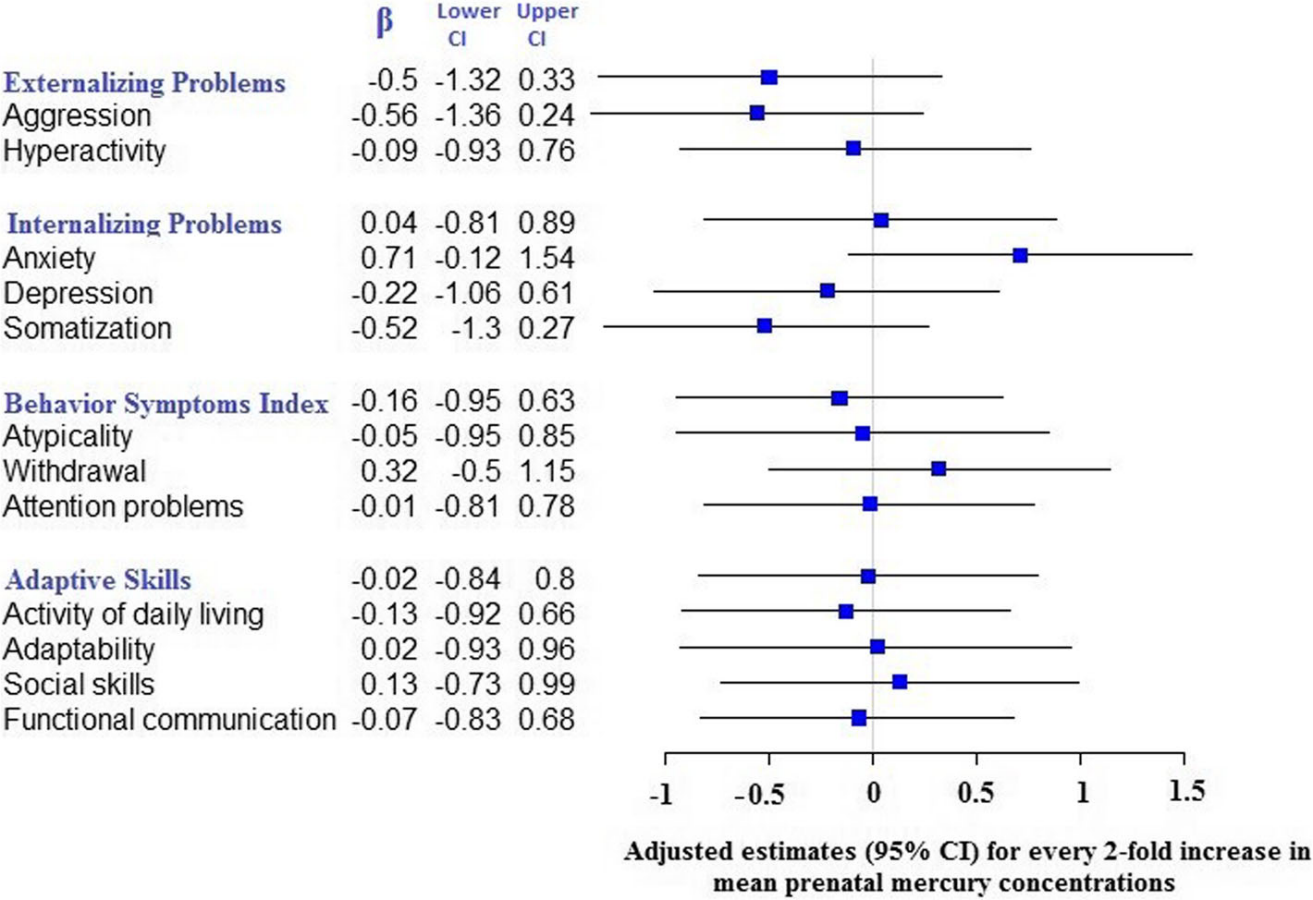
Adjusted associations (95% CI) in BASC-2 scores for every 2-fold increase in mean prenatal mercury concentrations through 2–8 years. The BASC-2 scales in “blue” color are composite scores. Mean prenatal mercury concentrations defined as a mean of maternal mercury concentrations at 16-weeks, 26-weeks of gestation, delivery, and cord blood mercury concentrations. Regression models were adjusted for fish intake, maternal age at delivery (≤ 30 years and > 30 years), maternal ethnicity (white and others), annual household income (> $40,000 and ≤ $40,000), maternal education (Completed Bachelor’s degree and not completed bachelor’s degree), marital status at baseline (Married/living with partner vs. unmarried/living alone), HOME score (≥ 40 or < 40), maternal depression during pregnancy (≤ 13 and > 13), child sex, child blood lead concentrations (mean of child blood lead levels at 12, 24, 36, 48, and 60 months), pre-natal serum cotinine concentrations (mean of 16 weeks, 26 weeks, birth and cord levels) and post-natal child blood mercury concentrations (mean of child blood mercury levels at 2, 3, 4, 5 years)

After adjustment for confounders, we did not find an overall significant association between mean prenatal mercury concentrations and BASC-2 composite scores or corresponding subscales through 8-years (Fig. 4). There was, however, a suggestion that prenatal mercury concentrations were associated with increased anxiety scores (β = 0.71, 95% CI = − 0.12, 1.54, *p* = 0.09) (Fig. 4).

In secondary analysis, we did not find any statistically significant associations between maternal mercury concentrations at 16- or 26 weeks of gestation, delivery and cord blood mercury concentrations and composite BASC-2 scores, attention problems, aggression, or hyperactivity subscales (data not shown). In contrast, we found a significant positive association between mercury concentrations at 16 weeks of gestation and BASC-2 anxiety subscales through 8-years (β = 0.83, 95% CI = 0.05, 1.62) (Table 3). However, the interaction between maternal mercury concentrations at 16-weeks of gestation and child sex was not statistically significant (*p* = 0.81).

**Table 3:** Adjusted^a^ associations (95% CI) between every 2-fold increase in mercury concentrations and BASC-2 anxiety scores through 8-years

Child sex	Time of measurement					
	Mean prenatal^b^	Mean prenatal excluding cord	16 weeks	26 weeks	At delivery	Cord
Total	0.71 (−0.12, 1.54)	0.82 (−0.04, 1.69)	**0.83 (0.05, 1.62)**	0.79 (−0.08, 1.67)	− 0.35^*^ (− 1.74, 1.03)	0.49 (− 0.24, 1.23)
Boys	0.80 (−0.24, 1.85)	0.76 (− 0.32, 1.85)	**1.22 (0.27, 2.16)**	0.64 (− 0.49, 1.77)	0.14 (−1.05, 1.32)	0.47 (− 0.55, 1.49)
Girls	0.91 (− 0.30, 2.12)	1.08 (− 0.20, 2.35)	0.70 (− 0.48, 1.87)	0.99 (− 0.28, 2.26)	**2.13 (0.85, 3.41)**	0.91 (− 0.14, 1.97)

The bold numbers [cells] indicate statistical significance at *p* < 0.05

^*^P value is 0.07 for the interaction term between mercury concentration at delivery and child sex

^a^Adjusted for fish intake, maternal age at delivery (≤ 30 years and > 30 years), maternal ethnicity (white and others), annual household income (> $40,000 and ≤ $40,000), maternal education (Completed bachelor’s degree and not completed bachelor’s degree), marital status at baseline (Married/living with partner vs. unmarried/living alone), HOME score (≥ 40 or < 40), maternal depression during pregnancy (≤ 13 and > 13), child sex, child blood lead concentrations (mean of child blood lead concentrations at 12, 24, 36, 48, and 60 months), pre-natal serum cotinine concentrations (mean of 16 weeks, 26 weeks, birth and cord concentrations) and post-natal child blood mercury concentrations (mean of child blood mercury levels at 2, 3, 4, 5 years)

^b^Mean prenatal mercury concentrations defined as a mean of maternal mercury concentrations at 16-weeks, 26-weeks of gestation, delivery, and cord blood mercury concentrations

We found that there was a statistically significant positive association (β = 2.13, 95% CI = 0.85, 3.41) between maternal mercury concentrations at delivery and BASC-2 anxiety subscales through 8-years in girls, but the association was not significant in boys (β = 0.14, 95% CI = − 1.05, 1.32). The *p*-value for the interaction term between maternal mercury concentrations at delivery and child sex was 0.07 (Table 3).

In secondary analyses, inclusion of other interaction terms for maternal race/ethnicity, child age, child blood lead concentrations, and maternal cotinine and PCB concentrations with mercury at each prenatal visit did not substantially alter our results.

**Fig. 5:**
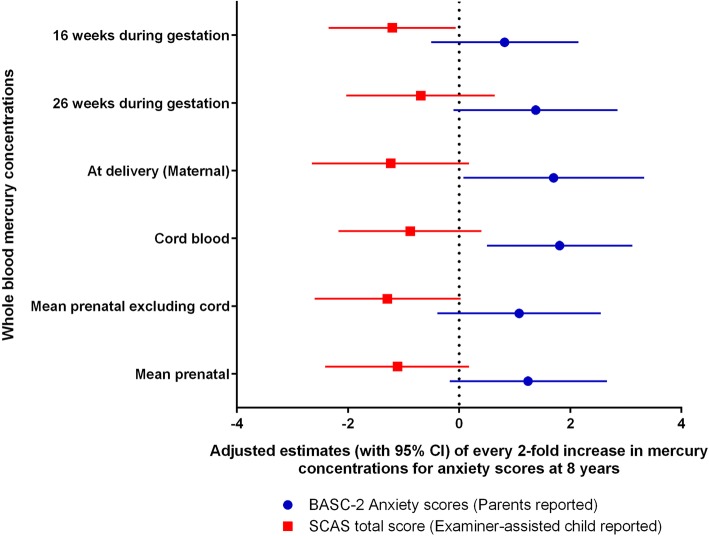
Adjusted regression coefficients for anxiety scores (95% CI) for every 2-fold increase in maternal whole blood mercury levels at 8 years of age. Mean prenatal mercury concentrations defined as a mean of maternal mercury concentrations at 16-weeks, 26-weeks of gestation, delivery, and cord blood mercury concentrations. Regression models were adjusted for fish intake, maternal age at delivery (≤ 30 years and > 30 years), maternal ethnicity (white and others), annual household income (> $40,000 and ≤ $40,000), maternal education (Completed Bachelor’s degree and not completed bachelor’s degree), marital status at baseline (Married/living with partner vs. unmarried/living alone), HOME score (≥ 40 or < 40), maternal depression during pregnancy (≤ 13 and > 13), child sex, child blood lead concentrations (mean of child blood lead levels at 12, 24, 36, 48, and 60 months), pre-natal serum cotinine concentrations (mean of 16 weeks, 26 weeks, birth and cord levels) and post-natal child blood mercury concentrations (mean of child blood mercury levels at 2, 3, 4, 5 years)

We observed a pattern of positive associations between maternal mercury concentrations and parent-reported BASC-2 anxiety subscale at 8 years. The association was statistically significant for cord blood (β = 1.81, 95% CI = 0.50, 3.12) and maternal (β = 1.70, 95% CI = 0.08, 3.33) mercury concentrations at delivery (Fig. 5). In contrast, there was a consistent pattern of *inverse* associations between prenatal mercury concentrations and self-reported SCAS total scores; the relationship with 16-week gestation mercury was significant (β = − 1.20, 95% CI = − 2.35, − 0.06) (Fig. 5). Parent-reported BASC-2 anxiety subscale and self-reported SCAS total score at 8-years were not correlated (Pearson *r* = 0.04, *p* = 0.60). We did not find any statistically significant association between prenatal mercury concentrations and other BASC composite scores/subscales at 8 years of age.

We did not find any significant association between postnatal child mercury concentrations and composite BASC-2 behavior scores or hyperactivity, attention, aggression, and anxiety subscales.

## Discussion

We did not find consistent evidence for adverse effects of very low-level, prenatal mercury exposure on behaviors among children in this cohort. There was some evidence that prenatal mercury concentrations were associated with increased parent-reported (BASC-2) anxiety scores from 2 to 5 years and at 8 years of age. However, we did not observe the same pattern of associations between prenatal mercury concentrations and child-reported anxiety scores (SCAS) at 8 years. Indeed, we found that maternal mercury concentrations during pregnancy were *inversely* associated with self-reported measures of anxiety using the SCAS in 8-year old children. Thus, while it is possible that prenatal mercury exposure is associated with higher anxiety scores in children, the inconsistent pattern we observed in this study suggests that this result could also be spurious.

We compared our findings for the prenatal mercury exposure and attention or externalizing (i.e., ADHD related) behaviors with other prospective cohort studies. In the Faroe Islands cohort, prenatal mercury exposure was significantly associated with impaired performance in attention at 7 and 14 years of age [10, 13]. However, the geometric mean cord blood mercury concentrations in the Faroe Islands cohort were about 31 times higher than those in the HOME Study cohort (22.5 μg/L vs. 0.71 μg/L) [10, 13]. In the Arctic Québec cohort, a significant positive association was found between cord blood mercury concentrations and teacher-reported attention problems, but the mean cord mercury concentrations were about 20- times higher than the mean mercury concentrations in our study (21.6 μg/L vs. 1.1 μg/L) [8]. Sagiv et al. [18] reported that prenatal mercury exposure was associated with ADHD related behaviors including inattention and hyperactivity/impulsivity. The mean maternal hair mercury levels in their study was 0.62 μg/g, which is equivalent to 2.5–3 μg/L in maternal blood and about 2.5 to 3 times higher than our study cohort [18, 21, 22]. Thus, our results may differ from earlier studies because the HOME Study cohort had very low levels of mercury exposure.

We also compared the findings of our study with other studies for children’s internalizing behaviors [8, 9, 15, 16]. In the Arctic Québec cohort, the association between cord blood mercury concentrations and teacher-reported internalizing problems was not statistically significant [8], but the authors only examined the effect of prenatal mercury exposure on composite internalizing problems; they didn’t examine the association of mercury exposure and anxiety. Ng et al. [16] did not find a significant association between cord blood mercury concentrations and internalizing behaviors until they stratified the results by apolipoprotein E (APOE) genotype carries. The authors found higher anxiety and internalizing problems scores with elevated cord blood mercury concentrations in APOE ε4 carriers compared with other APOE variants. The mean concentration in this cohort was also approximately 13.4 times higher than our study (14.7 μg/L vs. 1.1 μg/L) [16]. The authors did not find an association of prenatal mercury concentrations with either social or internalizing behaviors in a large birth cohort study conducted in Seychelles Islands, but they did not adjust for fish intake [9, 15]. This might bias the results towards the null as fish intake is often reported as a negative confounder for neurotoxic effects of mercury [35]; indeed, in a subsequent study, the investigators concluded that the beneficial effects of long-chain polyunsaturated fatty acids, present in fish, can obscure the neurobehavioral effects of mercury in longitudinal studies [20].

The United States Environmental Protection Agency derived a blood reference dose (RfD) for mercury of 5.8 μg/L [12, 18, 36]. Sagiv et al. [18] reported an apparent threshold level of 1 μg/g in maternal hair mercury concentrations at delivery for externalizing behaviors, which is approximately equivalent to 4–5 μg/L in maternal blood [21, 22]. The mean maternal blood mercury concentration at delivery in our study cohort is approximately 4 to 5 times lower than their reported threshold level of 1 μg/g. Thus, our findings are consistent with the findings of Sagiv et al. [18], who suggested there may be a threshold level for externalizing behaviors. Still, we found some evidence of an association between mercury exposure during early pregnancy and higher parent-reported anxiety scores that deserves further scrutiny. However, it will be challenging to disentangle the effect of low-level mercury exposures at different times during pregnancy given the high correlation between blood mercury levels over the course of pregnancy (Pearson *R* > 0.73).

Paradoxically, we found that maternal prenatal mercury concentrations were associated with higher parent reported BASC-2 anxiety subscales, but lower self-reported SCAS anxiety subscales in 8-year old children. Moreover, parent reported BASC-2 anxiety subscale at 8-year and self-reported SCAS total score at 8-year were not correlated with each other (Pearson *r* = 0.04, *P* = 0.60). The lack of a correlation between parent and child reported anxiety in our study was consistent with reports by other investigators [37–39]. For example, there was no significant relationship between SCAS and mothers’ reported anxious/rating subscale of the Child Behavior Checklist [39]. Although the parallel versions of the same anxiety instrument were used, parents and their children reported scales were only weakly correlated [39]. In another study, the authors found parents and child reported total score of Screen for Child Anxiety Related Disorders (SCARED), an instrument used to screen for childhood anxiety disorders, were modestly correlated (*r* = 0.30) [37]. These studies raise questions about whether parent or self-reported behavioral outcomes are more important for assessing child anxiety, especially in 7 to 8-years old children. It has been argued that self-report is an important method of assessing adolescent internalizing behaviors such as anxiety because emotional experiences are not always visible to others [39]. However, self-reported behaviors at 8 years of age could be less reliable. So, the results of the association between higher prenatal mercury exposure and lower self-reported anxiety levels should be interpreted with caution.

Several limitations should be considered while interpreting the findings of the study. First, we measured total blood mercury concentrations. Total blood mercury concentrations in our study participants, which were below 4 μg/L (mean = 0.95 μg/L), are a measure of both inorganic mercury and methylmercury [40–42]. Second, although we could control for an extensive number of potential confounders in this study, residual confounding due to unknown, unmeasured, or misclassified confounders cannot be ruled out. For instance, we measured prenatal mercury concentrations in mothers and they were also a part of parents-reported children behaviors. It is possible that the mercury exposure might have affected mother’s perceptions and interpretation of child’s behaviors. We attempted to address this by adjusting for maternal depression, however, it could have been affected through other pathways such as maternal anxiety. This would have biased our result either towards or away from the null [43]. Third, we conducted multiple statistical analyses. Finally, we had diminished statistical power to detect associations when we stratified our results by child sex, maternal ethnicity, PCBs, lead, cotinine, and age. Fourth, 320 dyads included in our study were statistically different in some maternal and child characteristics from the 69 dyads not included due to missing either mercury or behavior assessment data, which may result in a selection bias in our study.

This study also has several strengths. First, the low blood mercury concentrations in the HOME Study allowed us to examine the effect of prenatal mercury exposure at levels below a previously reported apparent threshold levels for children’s behavior. This is the first study to examine the effect of such a low-level prenatal mercury concentrations on a full spectrum of child behaviors. Second, mercury concentrations of our study cohort are comparable with the pregnant women in the NHANES study, a nationally representative sample of the U.S. population [44]. The geometric mean of total mercury concentrations in pregnant women was 0.67 μg/L in NHANES data of 2003–2004 (compared to 0.71 μg/L) [44]. Third, we had serial measures of mercury exposure during gestation, which allowed us to test for the windows of heightened vulnerability during fetal brain development. Fourth, we followed the children until 8 years of age so we could examine if mercury concentrations were associated with behaviors in older children.

## Conclusions

We did not find a consistent association between very low-level prenatal mercury exposure and behavior problem scores in children, but we did find some evidence of an association between very low-level mercury exposure during early pregnancy and parent-reported anxiety scores in children. The association of very low-level mercury exposures during early brain development with the development of anxiety and other behavior problems in children deserves further scrutiny.

## Abbreviations

ADHD: Attention Deficit Hyperactivity Disorder
APOE: Apolipoprotein E
BASC-2: Behavior Assessment System for Children – Second Edition
BDI-II: Beck Depression Inventory-II
BPA: Bisphenol A
BSI: Behavior Symptoms Index
CI: Confidence interval
HOME: Health Outcomes and Measurement of the Environment
LOD: Limit of Detection
PCBs: Polychlorinated biphenyls
RfD: Reference dose
SCAS: Spence Children’s Anxiety Scale
SD: Standard deviation
USCDC: United States Centers for Disease Control and Prevention
